# Bivalent Vaccine against *Streptococcus agalactiae* and *Aeromonas hydrophila* in Nile Tilapia (*Oreochromis niloticus*): A Laboratory-Phase and Large-Scale Study

**DOI:** 10.3390/ani13213338

**Published:** 2023-10-26

**Authors:** Açucena Veleh Rivas, Angelo Gabriel Vidal dos Santos, Adrieli Barboza de Souza, Gilson Bueno Junior, Gabriela Fernandes de Souza, Estevam Martins de Souza, Louisiane de Carvalho Nunes, Kelvinson Fernandes Viana

**Affiliations:** 1 Vaccine Development Technology Laboratory, Latin American Institute of Life and Nature Sciences, Federal University of Latin American Integration, Foz do Iguaçu 85870-650, Brazil; acucenarivas@gmail.com (A.V.R.); angelogvidal@outlook.com (A.G.V.d.S.); adrieli.souza@aluno.unila.edu.br (A.B.d.S.); gilson.bueno@unila.edu.br (G.B.J.); 2 Alquimia Pescados, Foz do Iguaçu 85870-180, Brazil; gabrielasouza.aquicultura@gmail.com (G.F.d.S.); estevampescador@gmail.com (E.M.d.S.); 3 Department of Veterinary Medicine, Agricultural Sciences and Engineer Center, Federal University of Espírito Santo, Alegre 29500-000, Brazil; louisianecn@gmail.com

**Keywords:** immunization, fish, aquaculture, tilapiculture, vaccine

## Abstract

**Simple Summary:**

Vaccination can prevent infection by opportunistic bacteria that affect fish. We have developed and analyzed a bivalent vaccine against two of the main pathogens that affect fish. We found that the vaccine was safe and effective in laboratory tests and in large-scale tests, with better survival and feed conversion in immunized animals. These results indicate the need for field tests to confirm real protection. This developed vaccine could allow fish farmers greater protection for commercial fish production.

**Abstract:**

One of the main factors limiting tilapia’s production is the occurrence of infections caused by *Aeromonas* and *Streptococcus* species. This work intended to evaluate a bivalent vaccine against *A. hydrophila* and *S. agalactiae* by intraperitoneal (i.p) administration in Nile tilapia (*Oreochromis niloticus*) in Brazil. The study was carried out in two phases: one in the laboratory, on a small scale, and from the results obtained, the study was expanded to a large scale in a production system in cages. The vaccine proved to be safe and effective in laboratory tests, with a vaccine efficacy (VE) of 93.66%. However, in large-scale tests with 12,000 tilapias, the VE was 59.14%, with a better food conversion ratio (1.54 kg) in the vaccinated group compared to the control group (1.27 kg). These results corroborate the efficiency of this tested vaccine; however, they indicate the need for field tests to attest to real protection.

## 1. Introduction 

Nile tilapia (*Oreochromis niloticus*) has a wide global distribution, present in more than 120 countries, and is one of the main fish species produced in the world [1]. This species is currently the most important fish farmed in Brazil, accounting for 63.93% (550,060 tons) of national fish farming [2]. The intensive production of Tilapia in cages presents advantages such as rapid implantation and investment return, high productivity, population control, and greater ease of handling [3]. However, the intensification of these production systems at high densities can alter water quality and the nutritional status of fish, contributing to chronic stress and immunosuppression of fish [4,5]. 

One of the main factors that limit tilapia production is the occurrence of opportunistic bacteria present in the water and in the fish microbiota. This can trigger diseases when the host is susceptible, with such bacteria being important pathogens with a significant economic impact on commercial fish production [6]. *Aeromonas* spp. and *Streptococcus* spp. are some of the main pathogens that affect fish. They can be diagnosed simultaneously, highlighting an association between these diseases [6,7,8].

The pathogenicity of *Aeromonas* spp. is associated with several virulence factors that aid in infection, allowing the invasion and colonization of other bacteria and causing damage [9]. In fish, *Aeromonas* spp. It mainly affects the liver, spleen, kidneys, brain, gills, and skeletal muscles, presenting hemorrhagic and ulcerative lesions, lack of appetite, lethargy, and hemorrhages, among other clinical signs, leading to death [7,9,10,11]. 

*Streptococcus agalactiae* (Lancefield’s group B Streptococcus) causes great losses in aquaculture in Brazil, especially serotype Ib, although strains of serotype Ia and III have been isolated in outbreaks [12], and is considered the main sanitary risk for commercial fish in the country [6]. Classic clinical signs of *S. agalactiae* infection in fish are anorexia, skin darkening; nervous symptoms associated with erratic swimming; lethargy; exophthalmia; corneal opacity; diffuse hemorrhages in the body, at the base of the fins, and at the opercula; ulceration of the epidermis; gill congestion; hepatomegaly; splenomegaly; and death [7,13,14]. There is also the occurrence of atypical clinical streptococcosis, where lethargic fish and outbreaks of deaths without external clinical signs are observed [15]. 

Currently, the main therapeutic measure used in bacterial outbreaks is the use of antibiotics, which, when used indiscriminately, cause important environmental impacts. Furthermore, there is the possibility of the selection and dissemination of resistant strains, as well as the presence of antimicrobial residues in fish and the environment [16,17,18,19]. 

An alternative to antibiotics in infection prevention is fish vaccination, which induces specific antibodies, conferring protection and safety to the immunized host [20]. Widely used due to economic benefits, inactivated cell vaccines are produced to protect fish against various bacterial diseases [21]. This work intended to evaluate the immunoprotective capacity of a bivalent vaccine against *Aeromonas hydrophila* and *Streptococcus agalactiae*, by intraperitoneal (i.p.) administration, in Nile tilapia (*Oreochromis niloticus*) from production tanks in Brazil.

## 2. Materials and Methods

### 2.1. Bacterial Strains

*Aeromonas hydrophila* and *Streptococcus agalactiae* were isolated from tilapias with clinical signs of bacteriosis from tanks in Foz do Iguaçu, Paraná, Brazil. For the isolation of vaccine and challenge strains, samples of the kidney, liver, spleen, heart, and brain were collected, inoculated in 5% sheep blood agar plate, and incubated at 30 °C for 48 h. After colony growth, these were identified through the Catalase [22] and Gram staining [23] tests and hemolytic activity [24] as in previous studies.

### 2.2. Biochemical Identification of Bacterial Strains

Automated bacterial identification was performed using VITEK^®^ 2 Compact equipment (bioMérieux, Inc., Foz do Iguaçu, Brazil) according to the manufacturer’s instructions, and for the identification of the strains, the GN card (card for the identification of Gram-negative bacteria) and the GP card (card for the identification of Gram-positive bacteria) were used [25]. *Streptococcus agalactiae* were subjected to serum agglutination analysis to determine the serotype with the commercial kit Strep B Latex Ib (Statens Serum Institut, Copenhagen, Denmark) lot LBIb-P1 and the serotype III with the commercial kit Strep B Latex III (Statens Serum Institut, Copenhagen, Denmark) lot LSBIII-1-8, according to the manufacturer’s recommendations [26]. Analyses were carried out by the Aquatic Animal Disease Laboratory at the Federal University of Minas Gerais. After identification, the strains were inoculated in a brain and heart infusion (BHI) and incubated in a shaker incubator at 30 °C for 48 h.

### 2.3. Molecular Identification of Bacterial Strains with Loop-Amplification-Mediated Purification (LAMP) 

For genomic material extraction, bacteria were cultivated for 12 h in a yeast extract medium (Kasvi, São José dos Pinhais, Brazil) and submitted to the extraction protocol of the Blood and Tissue Genomic DNA Miniprep System kit (Viogene Biotek, Taipei, Taiwan), according to the manufacturer’s instructions [27]. The obtained material was stored at −80 °C.

The LAMP reactions for *S. agalactiae* contained 1.6 μM of each primer FIP and BIP, 0.2 μM of each primer F3 and B3, 0.4 μM of each primer LF and LB, 1× reaction buffer termopol (20 mM Tris-HCl pH 8.8, 0.1 M KCl, 10 mM (NH_4_)_2_SO_4_, 2 mM MgSO_4_) (Cellco, São Carlos, SP, Brazil), 0.8 M of betaine (Sigma-Aldrich, St. Louis, MO, USA), 6 mM MgSO_4_, 2 mM dNTPs mix (Sigma-Aldrich, USA), 8 U Bst DNA polymerase (Cellco, São Carlos, Brazil) and 100 ng of DNA, in a total volume of 25 μL. The reaction was incubated for 60 min at 60 °C and then stopped at 90 °C for 2 min.

*A. hydrophila* analyses were carried out in a mix of 25 μL containing 1.6 μM of each primer FIP and BIP, 0.2 μM of each primer F3 and B3, and 0.4 μM of each primer LF and LB, 1× reaction buffer termopol (20 mM Tris-HCl pH 8.8, 0.1 M KCl, 10 mM (NH_4_)_2_SO_4_, 2 mM MgSO_4_) (Cellco, São Carlos, Brazil), 1.6 M of betaine (Sigma-Aldrich, USA), 6 mM MgSO_4_, 2 mM dNTPs mix (Sigma-Aldrich, USA), 8 U Bst DNA polimerase (Cellco, São Carlos, Brazil), and 100 ng of DNA. The reaction was incubated for 60 min at a temperature of 63 °C and then stopped at 90 °C for 2 min. The specific primers used for the detection of *A. hydrophila* (Cai, Y. et al. 2016 [28]) and *S. agalactiae* (Zhou, Q. et al., 2020 [29]) are shown in Table 1.

### 2.4. Development of the Bivalent Vaccine

For vaccine preparation, the cultures were inactivated by the addition of 10% buffered formalin, to a final concentration of 3%, in a shaker incubator at 100 RPM (rotations per minute) for 24 h at 25 °C. An aliquot of each culture was seeded in blood agar to confirm cell inactivation. Inactivated cultures were centrifuged at 6000 RPM for 30 min at 4 °C. The culture pellets were resuspended in sterile saline (NaCl 0.9%), and cells were counted in Neubauer chambers (Olen K5-0111, Kasvi Brazil), corresponding to a final concentration of 2 × 10^8^ cells/mL for *S. agalactiae* and 9 × 10^8^ cells/mL for *A. hydrophila.* For the vaccine dose, the concentration of each strain was adjusted to 1 × 10^7^ cells/dose, which was mixed with water-in-oil emulsions for intraperitoneal injection adjuvant (Montanide™ ISA 763 A VG, SEPPIC, Puteaux, France) [30]. After vaccine homogenization, it had its pH adjusted and was refrigerated for 48 h for stability analysis. The non-phase separation of the vaccine was stable. The stability analyses were followed for 12 months at temperatures between 2 °C and 8 °C.

### 2.5. Pre-Experimental Period

For initial analyses, in aquariums, the experiment was conducted at the Vaccine Production Technology Laboratory of the Federal University of Latin American Integration—UNILA—Brazil. Simulating high-stocking-density production, a total of 72 male fish (*Oreochromis niloticus*) with an average weight of 50 g of commercial origin from the cities of Toledo and Foz do Iguaçu, Paraná, were distributed in two aquaria of 200 L each, with a controlled temperature of 30 ± 2 °C [31,32,33]. The fish were fed twice a day with commercial food (36% gross protein, extruded) in a proportion of 3% live weight per day [34]. The aquariums contained 200 L of dechlorinated water, with a flow of 1000 L/h, continuous aeration, and cleaning performed daily by suction. Aquarium water was analyzed and corrected daily for ideal quality parameters such as pH, ammonia, nitrate, and dissolved oxygen.

### 2.6. Laboratory Experiment Design 

For vaccination and challenge, 36 individuals in the control group (non-vaccinated) and 36 individuals in the treatment group (vaccinated) were used, totaling 72 animals. In both procedures, the fish were anesthetized with eugenol (175 mg L^−1^) [35] and weighed. In the treatment group, each fish received, via i.p. administration, 50 μL of the vaccine. In the control group, each fish received, via i.p. administration, 50 μL of sterile saline (NaCl 0.9%). Induction of the experimental infection was performed after 30 days of vaccination; the concentration of both bacteria was adjusted to 1 × 10^8^ cells/dose, and doses of 50 μL were applied via i.p. administration. An agglutination test was conducted 30 days after the vaccination of the aquarium fish. Slide agglutination tests were conducted by mixing a drop of each antigen suspension of both bacteria, *A. hydrophila* and *S. agalactiae*, separately with a drop of the antiserum (NaCl 0.9% at 1:10, 1:100, and 1:1000 dilutions) of 3 fish from each laboratory experiment group on a glass slide [36]. Microscope-visible agglutinations were recorded as positive. Evaluation of clinical signs and fish mortality was performed daily in the post-vaccine and post-challenge periods by observing the first appearance of macroscopic external changes, behavioral changes, and deaths, which were noted in a specific form. Skin fragments were collected from the intracoelomic region of 5 fish from each experimental group one week after vaccination (T1), from 3 fish from each group two weeks after vaccination (T2), and from 2 fish from each group three weeks after vaccination (T3). At the end of the experiment, all surviving fish were euthanized by deepening the anesthetic plane and necropsied, and liver and spleen fragments were collected from 4 fish from each experimental group. The samples were placed in paraformaldehyde and refrigerated until the time of histopathological analysis. The samples were sent to the Federal University of Espírito Santo (UFES) and processed for histological sections of 5 μm, stained with hematoxylin and eosin. The slides were analyzed for structural and cellular alterations and classified according to the intensity of the lesion: (0) absent, (1) slight, (2) moderate, and (3) intense or severe.

### 2.7. Field Experiment Design 

For field analyses, 12,000 fish were selected, divided into two groups, a control group (6000) and a vaccinated group (6000), and distributed in 10 m^3^ cages with a population density of 150 animals/m^3.^ The animals had an initial weight between 80 g and 120 g. Feeding was performed twice a day using commercial feed. The vaccine evaluation in the field was carried out by submitting the animals to field conditions at high density without experimental challenge, only natural infection. For vaccination, the animals were anesthetized with eugenol (175 mg L^−1^) [35]. The vaccine was administered via intraperitoneal injection, applying 50 μL per fish. The control group received no treatment. The biometrics were performed within a 30-day interval to estimate the animals’ biomass. Fifty animals per cage were collected and weighed to assess the average mass of the animals. In addition, all animals present in each tank were weighed to assess the biomass of each group throughout the study.

### 2.8. Statistical Analysis

To verify the statistical significance between the vaccinated and control groups in the laboratory and field experiments, the Yates-corrected chi-square test (*p* < 0.05) was used. The relative risk (RR) (CI = 95%) was calculated to verify the strength of the association between exposure to the vaccine and its protective effect. To evaluate the weights of the control and vaccinated groups in the field, a Welch’s test (*p* < 0.05) was applied. The Kaplan–Meier curve was used to analyze the effect of vaccination and for the graphic representation of the probability of survival. Once the proportionality of the risks was verified, the Cox semi-parametric model was used to compare the experimental groups. To evaluate the equality of the survival functions of the groups, the log-rank test (*p* < 0.01) was used. For the calculations mentioned, the GraphPad Prism 10 and the PAST (4.03) software were used. Vaccine efficacy was calculated from the formula VE = (1 − OR) × 100, with OR being the odds ratio value.

## 3. Results

### 3.1. Bacterial Identification of Aeromonas hydrophila and Streptococcus agalactiae

The biochemical identification obtained a 99% probability for *A. hydrophila* and a 98% probability for *S. agalactiae*. Serotyping analysis showed that the isolated *S. agalactiae* refers to serotype III.

**Figure 1:**
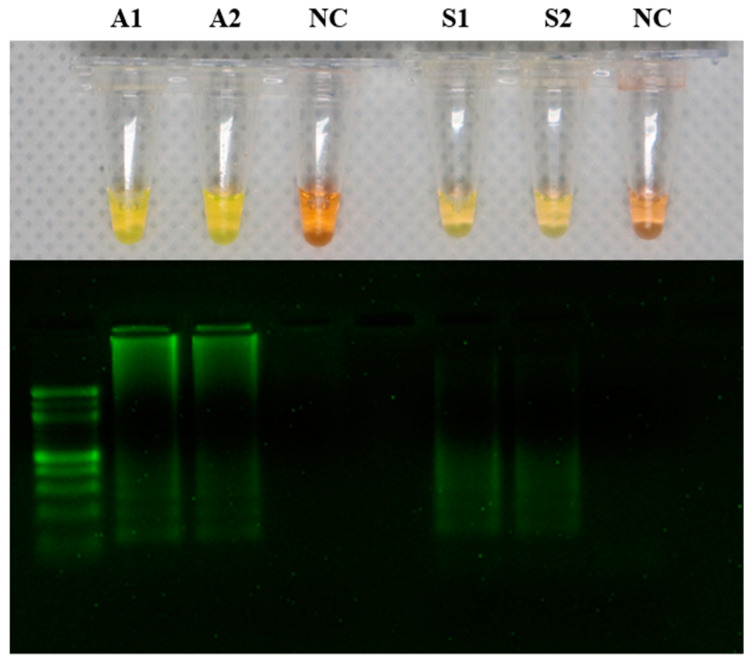
Detection of DNA amplification in the loop-mediated isothermal amplification (LAMP) assay by a change in color from orange to yellow. LAMP results in addition of SYBR green dye showing A1 and A2 as positive for *Aeromonas hydrophila*, S1 and S2 as positive for *Streptococcus agalactiae*, and NC (negative control) as a negative reaction. The LAMP reaction product was analyzed on a 2% agarose gel.

The LAMP products were detected by adding SYBR green fluorescence dye. The tubes containing *A. hydrophila* samples (A1 and A2) and the tubes containing *S. agalactiae* samples (S1 and S2) produced positive reactions that appeared yellow, while the negative reaction solution remained orange. The LAMP products were visualized using two percent agarose gel electrophoresis (Figure 1).

### 3.2. Vaccinated Group Fish Antiserum Presented Antigen Agglutination after Vaccination in Laboratory Experiment

**Figure 2:**
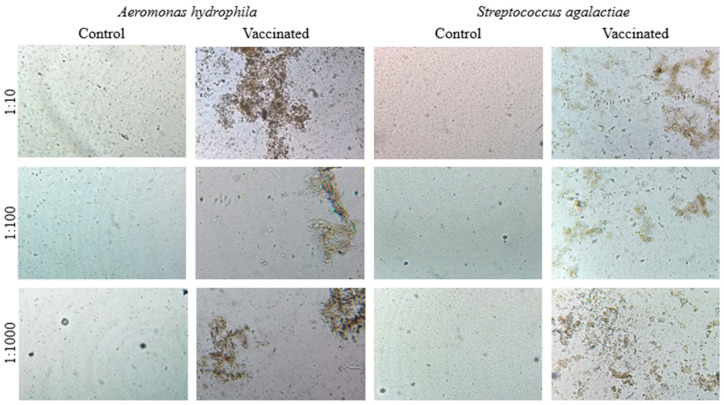
Photomicroscopy of the direct agglutination test with Nile tilapia sera from the control and vaccinated groups at dilutions of 1:10, 1:100, and 1:1000 associated with inactivated *Aeromonas hydrophila* and *Streptococcus agalactiae* antigens.

The agglutination tests showed strong agglutination with vaccinated fish sera for the antigens of both bacteria in the three dilutions tested (1:10, 1:100, and 1:1000) after 30 days of vaccination by i.p. administration. Fish sera from the control group showed no agglutination for the antigens of both bacteria (Figure 2).

### 3.3. Histopathological Analysis of Liver, Spleen, and Tissue from the Vaccine Injection Region

**Figure 3:**
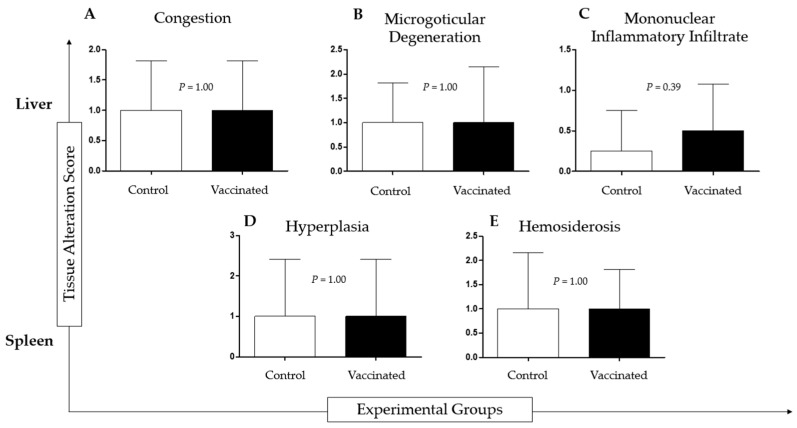
Histopathological analysis of liver and spleen of vaccinated and non-vaccinated (control) Nile tilapia after intraperitoneal infection with 1 × 10^8^ cells/dose of *Aeromonas hydrophila* and 1 × 10^8^ cells/dose of *Streptococcus agalactiae* during the experimental period of 30 days. The groups did not show significant differences.

Histopathological analysis of tilapia livers from both groups showed congestion, diffuse or multifocal microgoticular degeneration, and diffuse or multifocal mononuclear inflammatory infiltrate. Analysis of the spleen of tilapia from both groups revealed white pulp hyperplasia (vaccinated group), red pulp hyperplasia (control group), and hemosiderosis. There was no statistical difference between the vaccinated and control groups. (Figure 3).

Tissue histological analysis of the intraperitoneal region of tilapia in the vaccinated group showed collagen deposition perpendicular to the musculature, focal areas of cartilage formation, hyperkeratosis and hyperplasia in T2, and subepithelial melanocytes in T3. The control group presented inflammatory infiltrate in T1 and hyperplasia, microgoticular muscle degeneration, and autolysis in T3.

### 3.4. Bivalent Vaccine against Aeromonas hydrophila and Streptococcus agalactiae Efficacy in Laboratory and Field Experiments

In the laboratory experiment, the bivalent vaccine against *A. hydrophila* and *S. agalactiae* inoculated via i.p. administration in tilapia obtained a chi-square test result with *p* = 0.0042, RR = 0.13 (95% CI: 0.02–0.75), and VE = 93.66%. In the field experiment, the bivalent vaccine obtained a chi-square test result with *p* = 0.0001, RR = 0.43 (95% CI: 0.36–0.50), and VE = 59.14% (Table 2).

### 3.5. Bivalent Vaccine against Aeromonas hydrophila and Streptococcus agalactiae Protects Tilapia against Clinical Signs of Both Diseases

**Figure 4:**
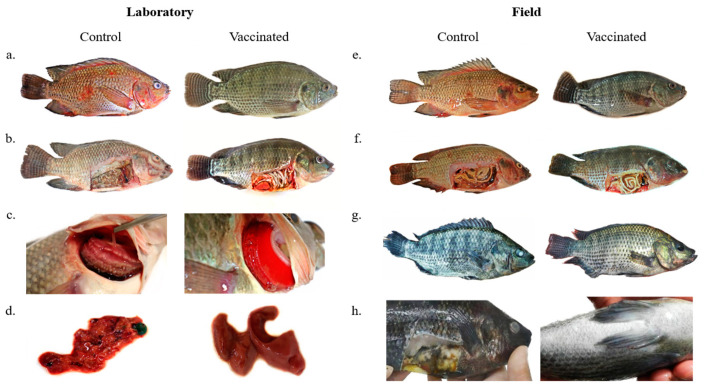
(**a**) Control group fish after experimental infection with *A. hydrophila* and *S. agalactiae* presenting body and fin hemorrhages and ulcerative lesions. Fish from the vaccinated group showing no external clinical signs after experimental infection. (**b**) Control group fish after experimental infection with *A. hydrophila* and *S. agalactiae* presenting skin pallor, yellowish fluid in the visceral cavity, and organ deterioration. Fish from the vaccinated group showing no internal clinical signs after experimental infection. (**c**) Control group fish after experimental infection with *A. hydrophila* and *S. agalactiae* presenting excess mucus, necrosis, and pallor of the gills. Fish gills of the vaccinated group with normal appearance and color after experimental infection. (**d**) Fish liver from the control group after experimental infection with *A. hydrophila* and *S. agalactiae* showing deterioration and hemorrhage. Fish liver of the vaccinated group showing normal appearance and color after experimental infection. (**e**) Control group fish presenting body and fin hemorrhages. Vaccinated group fish showing no external clinical signs. (**f**) Control group fish presenting skin pallor, yellowish fluid in the visceral cavity, and organ deterioration. Vaccinated group fish showing no internal clinical signs. (**g**) Control group fish presenting tail rot and corneal opacity. Vaccinated group fish showing weight gain and no clinical signs. (**h**) Control group fish showing skin darkening, corneal opacity, and internal organ deterioration.

In the post-vaccinated period in the laboratory experiment, fish of both groups presented low food intake, which returned to normal on the next day. At the end of the experiment, the vaccinated group of fish that were necropsied did not show macroscopic external and internal clinical signs. External clinical signs of control group fish were hemorrhages; deterioration and hemorrhage of fins; ulcerative lesions; anorexia; altered skin color; gills with excess mucus and presence of necrotic areas (yellowish or brown); lethargy; erratic swimming (indicating central nervous system involvement); and opaque eyes. Internal clinical signs of fish in the control group were yellow or bloody fluid present in the visceral cavity; a decayed and hemorrhagic liver; a dark gallbladder; and a pale color of organs (Table S1) (Figure 4).

### 3.6. Mortality Was Lower in the Vaccinated Tilapia Groups after Laboratory Experiment Challenge and Lower in the Field Experiment without Challenge

**Figure 5:**
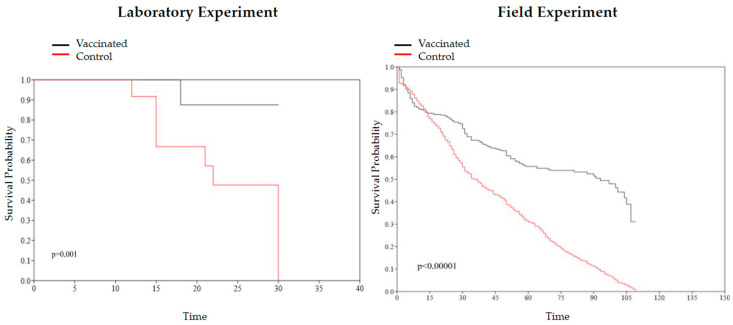
Kaplan–Meier survival curve and log-rank test comparing the vaccinated and control groups from laboratory (*p* = 0.001) and field (*p* < 0.00001) experiments.

Mortality in the vaccinated group of the laboratory experiment was 01 (2.94%) and in the control group was 11 (32.35%). In the vaccinated group, the fish died on the 18th day after experimental infection but did not present clinical signs of the diseases (only signs of fight). In the control group, the 11 fish died on days 12, 15, 21, 22, and 30 after the experimental infection. Survival curves of the control and vaccinated groups in the laboratory experiment had a significant difference (*p* = 0.001), and Cox’s analysis showed a significant risk of event (death) between groups, with a reduction in the risk of death in the vaccinated group: HR = 0.06056 (95% CI: 0.003256–0.3252) (Figure 5).

In the field, the vaccine was able to reduce mortality; control and vaccinated groups had a mortality rate of 7.78% and 3.33%, respectively. In addition, the daily mortality rate was reduced to less than half in the vaccinated cages. Survival curves of the control and vaccinated groups in the laboratory experiment had a significant difference (*p* < 0.00001), and Cox’s analysis showed a significant difference, with a reduction in the risk of death in the vaccinated group: HR = 0.1713 (95% CI: 0.1281–0.2244) (Figure 5).

### 3.7. Effect of Vaccination on Weight Gain and Feed Conversion

In the laboratory experiment, on the vaccination day, fish of the control and vaccinated groups had an average weight of 50.2 ± 17.2 g and 50.0 ± 19.2 g, respectively, with no significant difference (*p* = 0.7133). Thirty days after vaccination and before experimental infection, the control and vaccinated fish had an average weight of 119.3 ± 27.9 g and 103.3 ± 23.7 g, respectively, with a significant difference (*p* = 0.0092). Sixty days after vaccination and thirty days after experimental infection, the control and vaccinated fish had an average weight of 150.0 ± 50.3 g and 164.2 ± 33.7 g, respectively, presenting no significant difference (*p* = 0.2197).

In field experiments, vaccinated animals had greater growth than the control animals, as shown in Table 3. Both groups showed a similar pattern of fattening during the first months; however, at the end, the vaccinated group had approximately 22% more body mass per animal compared to the control. Added to this, a greater homogeneity was also observed in the final average weight of the animals in the vaccinated cages. The feed conversion ratio (FCR) considers the amount of food given to the animals and the final biomass. The analysis of this parameter showed that the immunized animals had better use of the food compared to the non-immunized ones, being able to transform the food more efficiently into body mass (Table 3).

## 4. Discussion

Inactivated whole-cell vaccines correspond to the most used type for veterinary vaccines, as they do not represent the risk of virulence reversion [37,38,39]. The intraperitoneal administration system presents better results compared to other systems, such as by immersion bath, via spray, or orally, since these systems, despite being easier to handle, do not effectively stimulate antibody production [38,39,40,41,42].

Studies showed a greater agglutination titer after fish vaccination against *Streptococcus* spp. and *Aeromonas* spp. [43,44,45,46]. In the present study, agglutination was shown to be increased for *A. hydrophila* and *S. agalactiae* in the three dilutions tested after 30 days of vaccination in the laboratory experiment, confirming that the vaccine induced the production of antibodies to both bacteria.

With the aim of mimicking the occurrence of bacterial outbreaks in production tanks where bacteria would replicate in large quantities, especially at high temperatures, the fish were challenged with a high dose, showing that the vaccine was able to protect the vaccinated group. However, despite mimicking high-density production in the laboratory, in addition to thermal stress, large-scale tests showed different effectiveness. However, when compared with the control group in the field, the vaccinated group showed an improvement in survival and feed conversion.

Another important factor in the use of intraperitoneal vaccines is cost-effectiveness, where the effectiveness is sufficiently high in relation to the costs of the vaccine and the labor required for individual vaccination of fish. The application of more than one vaccine dose can increase protection in fish [47,48]. However, the investment required to repeat the dose may not be attractive to the producer due to logistical problems and the cost of commercial production. The bivalent vaccine was able to provide protection to tilapia vaccinated with just one dose, representing a good cost–benefit ratio.

Used in the field, the bivalent vaccine proved to be effective in reducing mortality in tilapia, and the VE obtained was 59.14%. Pasnik (2005) [49] showed that the immune response generated is effective 180 days after vaccination, being able to protect animals throughout the fattening period with a single dose. This study managed to keep the mortality rate reduced over 118 days, showing that vaccination with one dose protects the animals during the entire fattening period. It is noteworthy that the above analyses were conducted with the experimental infection of the animals. In this work, the animals were submitted to conditions of high production density to intensify the natural infection rate.

The bivalent vaccine incorporated with the adjuvant proved to be non-toxic to fish in the vaccinated group, proving its safety. Histopathological analysis of the skin, liver, and spleen of both vaccinated and control groups showed no significant differences. Steckert et al. (2018) [50] concluded that alterations such as hyperplasia may be present in non-diseased animals, suggesting an adaptation of fish to the confinement environment. The skin of fish in the control group showed a mononuclear inflammatory infiltrate, characterized by a large amount of leukocytes accumulated in an inflammatory response site in the first week after the inoculation of sterile saline solution, hyperplasia, and macro- and microgoticular degeneration after three weeks. The histological findings of the fish skin of the vaccinated group showed that 2 to 3 weeks after immunization, the vaccine inoculation site underwent a healing process. This factor is extremely important, since a vaccine candidate should not leave lesions in the inoculum region except in the first days after vaccination due to the expected inflammatory process.

In large-scale trials, vaccination was shown to help increase productivity and improve feed conversion. Immunized fish, after 118 days of the experiment, weighed about 0.792 kg (±0.016) compared to the 0.657 kg (±0.10) in control. This indicated an increase in biomass and greater homogeneity due to immunization. The better use of the feed can be related to the reduction in stress inherent to the presence of infections. In addition, most of the fish in the control group had several lesions on the scales, and when analyzing practical and legal issues under production conditions in the country, fish with skin lesions cannot be marketed [51].

## 5. Conclusions

In general terms, immunization with a bivalent vaccine against *A. hydrophila* and *S. agalactiae* administered via i.p. administration demonstrated to be safe and effective in reducing mortality in fish raised in high-density production tanks. Furthermore, the benefits of vaccination also led to an improvement in the animals’ feed conversion, representing direct gains in fish productivity.

## 6. Patents

Patent of invention titled “Bivalent Vaccine for Tilapia” (BR1020190263644), deposited in Instituto Nacional de Propriedade Intelectual (INPI).

## Figures and Tables

**Table 1:** Primers for detection of *A. hydrophila* and *S. agalactiae* in molecular identification.

Species	Primers
*A. hydrophila*	F3 ATATGATGCGCTTGAGCC
	B3 ACCACCGTTATTGATGACTG
	FIP GAGCAGCATTTGCATTAGCAACATATTTTGATGCTGAGACAATGACAC
	BIP ACATCCTGAAATTGGAGAAGACTTTTTTCCTGACGAATATCTTCTGGAAT
	LF TGCATGGTGCTTATCATGATGT
	LB AGGCGCTCTTAGCTGATGT
*S. agalactiae*	F3 CCGAAGGTGTTCCACTTCC
	B3 ATAACGGCAATCAGACCTTC
	FIP GCTGCGGAATGTTGTTGGTGTTTTGGTGTGGAAGTAGCGATG
	BIP ACCTGGTGGGCTTCCGTATTTTGCCTTCTTGCTGTAGTCC
	LF CCTGATAGGCGTCGTTCC
	LB CCGTACTCTGAACTCCTACATG

**Table 2:** Efficacy of a bivalent inactivated vaccine against *Aeromonas hydrophila* and *Streptococcus agalactiae* inoculated via i.p. administration in Nile tilapia (*Oreochromis niloticus*). VE = Vaccine Efficacy.

Experiment	Treatment	Deaths/Total (%)	*p* < 0.05	VE%
Laboratory	Vaccinated	1/34 (2.94)	0.0042 *	93.66
	Control	11/34 (32.34)		
Field	Vaccinated	200/6000 (3.33)	0.0001 *	59.14
	Control	467/6000 (7.78)		

* Chi-square corrected by Yates.

**Table 3:** Final Average Weight, Food Conversion Ratio (FCR), and Average daily mortality of Control and vaccinated groups in field experiment.

	Final Average Weight (kg)	FCR (kg)	Average Daily Mortality
Control	0.657 (±0.10)	1.54	5.12 (±3.16)
Vaccinated	0.792 (±0.016)	1.27	2.40 (±4.37)

## Data Availability

No new data were created or analyzed in this study. Data sharing is not applicable to this article.

## Supplementary Materials

The following supporting information can be downloaded at: https://www.mdpi.com/article/10.3390/ani13213338/s1, Table S1: External and internal physical characteristics and behavior characteristics analyzed in the post-vaccination and post-infection periods and in necropsy, in control and vaccinated groups. dpv = days post-vaccination; dpi = days post-infection; C = control group; V = vaccinated group.
