# Supplementary material for: Bivalent Vaccine against Streptococcus agalactiae and Aeromonas hydrophila in Nile Tilapia (Oreochromis niloticus): A Laboratory-Phase and Large-Scale Study
Source: Animals (Basel). 2023 Oct 26;13(21):3338. doi: 10.3390/ani13213338 (PMC10650067; doi:10.3390/ani13213338)
Supplement: Supplementary file 1 [file animals-13-03338-s001.zip › animals-2588896-supplementary.pdf]

Table S1. External and internal physical characteristics and behavior characteristics analyzed in the post-vaccination and post-infection periods and in necropsy, in control and vaccinated groups. dpv = days post-vaccination; dpi = days post-infection; C = control group; V = vaccinated group.

**Clinical signs**	**1 dpv**	**1 dpi**	**4dpi**	**5 dpi**	**6 dpi**	**7 dpi**	**12 dpi**	**15 dpi**	**17 dpi**	**18 dpi**	**20 dpi**	**21 dpi**	**22 dpi**	**29 dpi**	**30 dpi**	**Necropsy**
Anorexia																C
Exophthalmos																
Abdominal distension													C			C
Erratic swimming									C							
Skin lesions			C	C	C	C							C			
Opaque eyes									C				C			
Skin darkening									C							
Lethargy											C			C		
Hemorrhage					C	C							C	C		
Hemorrhagic lesions													C	C		
Fins Erosion									C					C		
Ulceration																
Peeling														C	C	
Low food intake	VC	VC													C	
Fluid in the visceral cavity																C
Gills necrosis																C
Alter in organ color																C
Alter in organ size																
Organ deterioration																C
Death							C	C		V		C	C		C	
